# The spatial and temporal dynamics of global meat trade networks

**DOI:** 10.1038/s41598-020-73591-2

**Published:** 2020-10-07

**Authors:** Min Gon Chung, Kelly Kapsar, Kenneth A. Frank, Jianguo Liu

**Affiliations:** 1 Center for Systems Integration and Sustainability, Department of Fisheries and Wildlife, Michigan State University, East Lansing, MI 48823 USA; 2 Sierra Nevada Research Institute, University of California, Merced, CA 95343 USA; 3 Department of Counseling, Educational Psychology and Special Education, Michigan State University, East Lansing, MI 48824 USA

**Keywords:** Complex networks, Sustainability

## Abstract

Rapid increases in meat trade generate complex global networks across countries. However, there has been little research quantifying the dynamics of meat trade networks and the underlying forces that structure them. Using longitudinal network data for 134 countries from 1995 to 2015, we combined network modeling and cluster analysis to simultaneously identify the structural changes in meat trade networks and the factors that influence the networks themselves. The integrated network approach uncovers a general consolidation of global meat trade networks over time, although some global events may have weakened this consolidation both regionally and globally. In consolidated networks, the presence of trade agreements and short geographic distances between pairs of countries are associated with increases in meat trade. Countries with rapid population and income growth greatly depend on meat imports. Furthermore, countries with high food availability import large quantities of meat products to satisfy their various meat preferences. The findings from this network approach provide key insights that can be used to better understand the social and environmental consequences of increasing global meat trade.

## Introduction

The trade of meat and meat products generates complex networks as economies and populations expand throughout the world^1–3^. Globally, rapid dietary transition toward increased meat consumption accelerates countries’ dependence on meat trade to fulfill increased domestic demands^4–6^. The dynamics of global meat trade networks increase interdependencies between sending countries (exporters, supply areas) and receiving countries (importers, demand areas), placing stress on both human and natural systems across the world. For example, meat production for export can result in land use change and biodiversity loss^7–9^. Likewise, diets high in meat acquired through trade are positively associated with rates of diet-related, non-communicable diseases worldwide^10–12^.

Since global meat trade networks vary according to diverse socioeconomic and environmental factors in both sending and receiving countries, the structural changes in the networks make it difficult to predict meat flows among countries^6,13,14^. For example, over the last two decades, the number of trade agreements between countries has rapidly increased due to economic and political benefits in the era of globalization^15^, but their impacts on the structure of global meat trade networks are unclear. It is also uncertain whether increases in meat trade contribute to enhanced food availability in low-income countries^16^. Furthermore, little research has quantified the dynamics of global meat trade networks and examined their underlying forces using a holistic framework and approach.

Network analysis provides a comprehensive way to quantify the network structures of meat trade over time and uncover underlying drivers in both sending and receiving countries. Over the past two decades, network models have been developed to explain network and temporal dependencies, as well as the drivers of network structures, based on statistical inference^17^. However, most food trade studies that use network approaches focus on identifying the structural characteristics of the networks such as betweenness, centrality, and clusters^18–21^, as opposed to the drivers that contribute to the structural changes of the networks over time.

To fill this research gap, we combined a network model approach with cluster analysis of meat trade in 134 countries from 1995 to 2015. The combination of the network modeling and cluster analysis uniquely identifies sub-networks within the global meat trade network while also determining how these sub-networks have changed over time. Our study answers two questions: (1) How has the network structure of meat trade changed over time? and (2) Which factors contribute to recent increases in meat trade? By estimating network models of global meat trade, this study provides insight into the fluctuations of global meat trade networks as a result of different political, socioeconomic, and environmental factors across the world. Furthermore, this study identifies countries that are responsible for increases in meat trade and determines the important factors influencing meat trade sub-networks using an integrated network approach.

## Results

### Structural change of global meat trade networks

**Figure 1:**
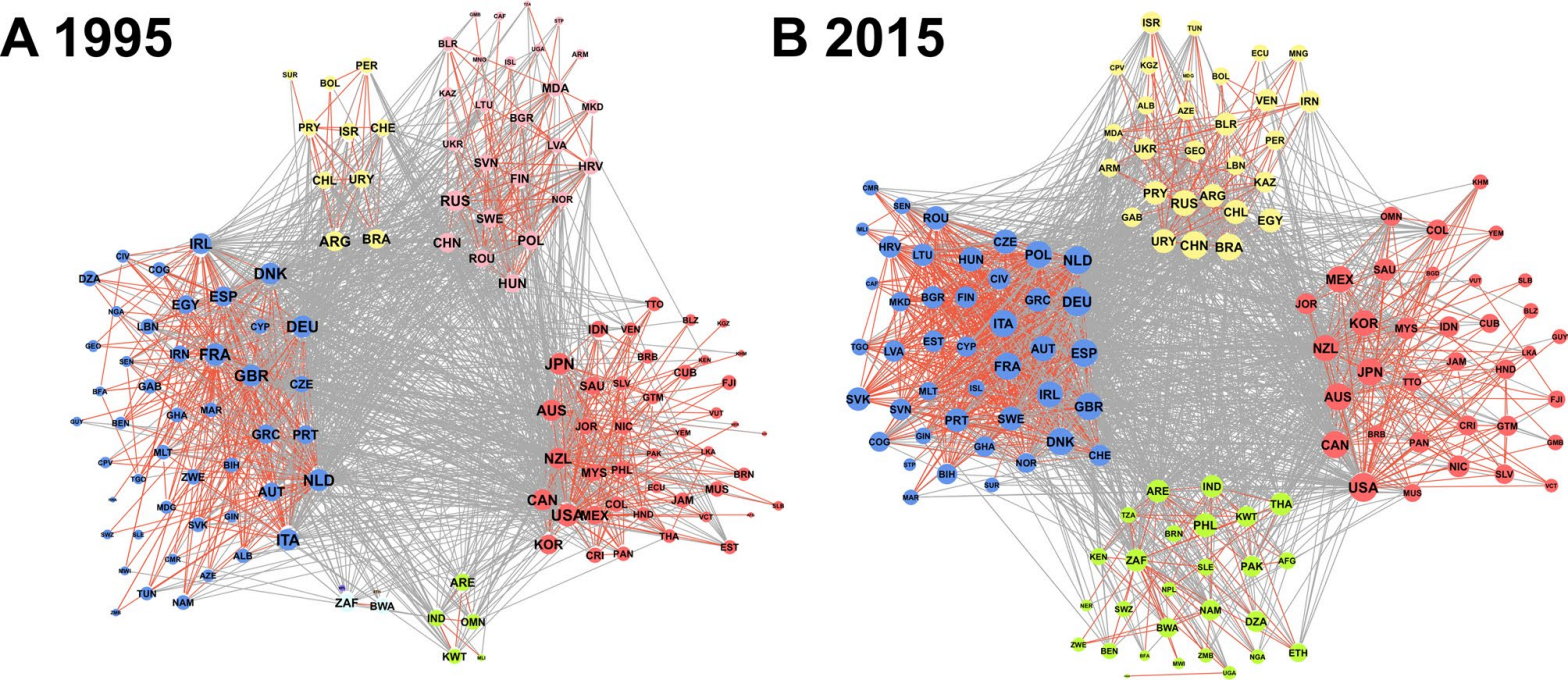
Clusters of global meat trade networks in **(A)** 1995 and **(B)** 2015. The size of actors represents the amounts of meat exports and imports. Red ties indicate meat trade flows in the same cluster, and gray ties indicate meat trade flows with other clusters. The core countries are located in the center of the network maps. The network graph was generated by R 3.6.2^22^.

In 1995, the global meat trade network had eight clusters, which were divided into four major clusters and four minor clusters (Fig. 1). Cluster results indicate that countries in the same cluster tended to export and import more meat products with each other than those in other clusters. Each major cluster included more than 10 countries, and the outcomes of the clustering algorithm indicated that these clusters were primarily based on geographic locations (Europe; North America and South Asia; East and North Asia; and South America) (Supplementary Fig. 1A). Combined, these major clusters included 125 out of the 134 total countries, including all high-income countries. Countries in the remaining minor clusters did not actively engage with global meat trade, were middle- and low-income countries, and were also grouped by geographic location.

Global meat trade networks have consolidated over time, particularly since 2004 (Supplementary Fig. 1J). Between 2004 and 2015, the network structure in each year had mainly three or four major clusters. The meat trade network in 2015 consisted of four major clusters (Europe; North America and South Asia; South America and North Asia; and the Middle East and Africa). These clusters included all 134 countries. Informed by different clustering algorithms, we confirmed the robustness of clusters in global meat trade networks in each year. The use of different clustering algorithms revealed that the major clusters remained between the optimal and the walktrap community algorithms (which had the first- and second-highest modularity values, respectively), with the exception of small and low-income countries (Supplementary Table 2 and 3).

**Figure 2:**
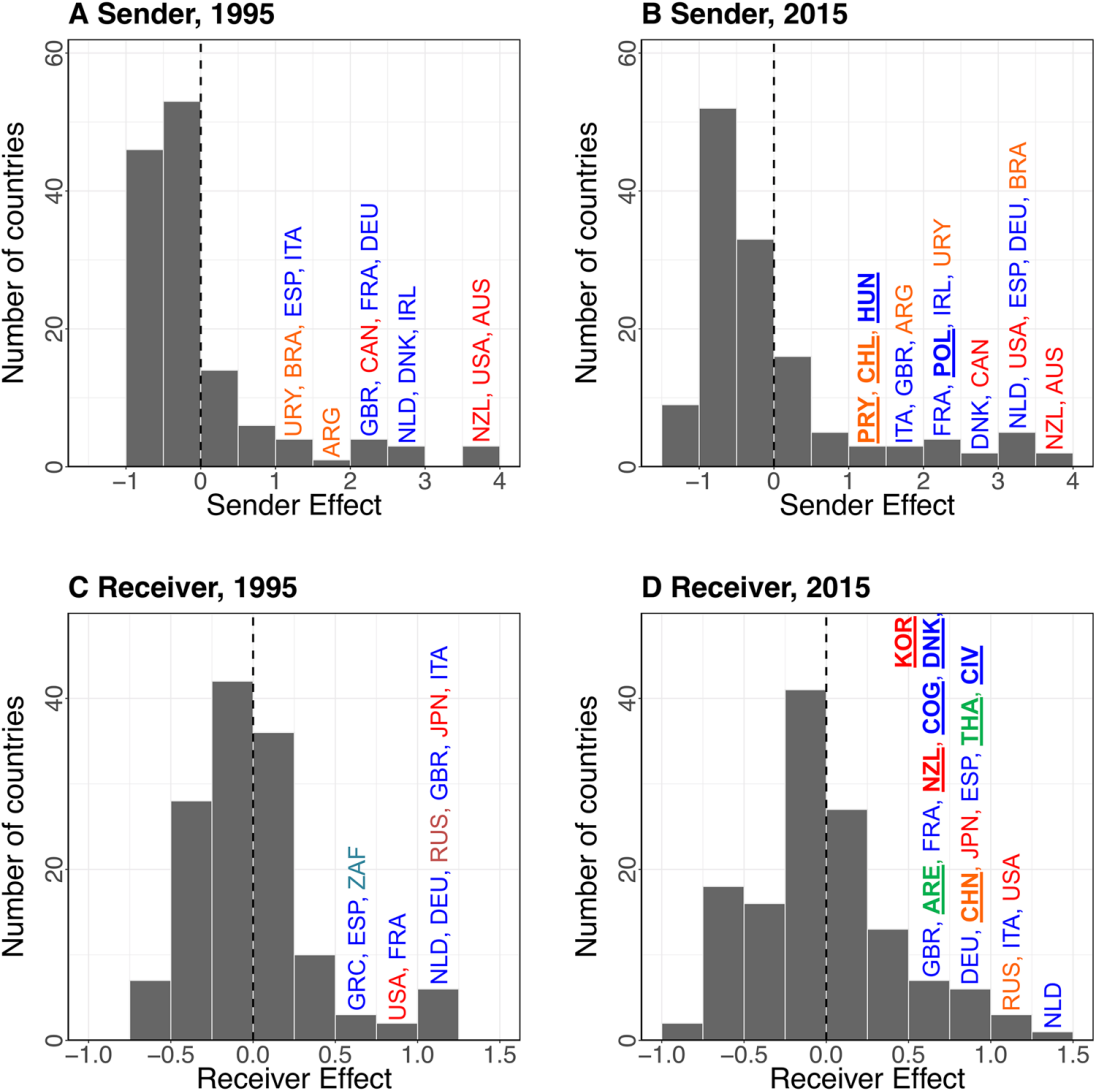
Distributions of **(A)** the sender effects in 1995, **(B) **the sender effects in 2015, **(C)** the receiver effects in 1995, and **(D)** the receiver effects in 2015.

A mixed-effects model allowed us to identify country-specific random effects for sending and receiving countries from 1995 to 2015 (Fig. 2). These random effects measured which countries played substantial roles in global meat trade networks. Over the period of 1995–2015, New Zealand and Australia were among the most active meat exporting countries, while the Netherlands and the United States were among the most active senders (exporters) and receivers (importers) in the network, simultaneously. This result is further reinforced by the presence of these most active senders and receivers in the center of our network maps (Fig. 1).

We also identified new active senders and receivers in each cluster, or sub-network, of the global meat trade network through integration with our cluster analyses (Fig. 1). Over the past two decades, Poland and Hungary (located in the blue cluster) and Paraguay and Chile (located in the yellow cluster) strengthened their roles as meat senders (Figs. 1 and 2). During the same period, China (located in the yellow cluster) and Cote d’Ivoire, Republic of the Congo, and Denmark (located in the blue cluster) became active meat receivers. South Korea and New Zealand (located in the red cluster) and Thailand and United Arab Emirates (located in the green cluster) also grew into active receivers in global meat trade networks.

### Factors affecting global meat trade

**Figure 3:**
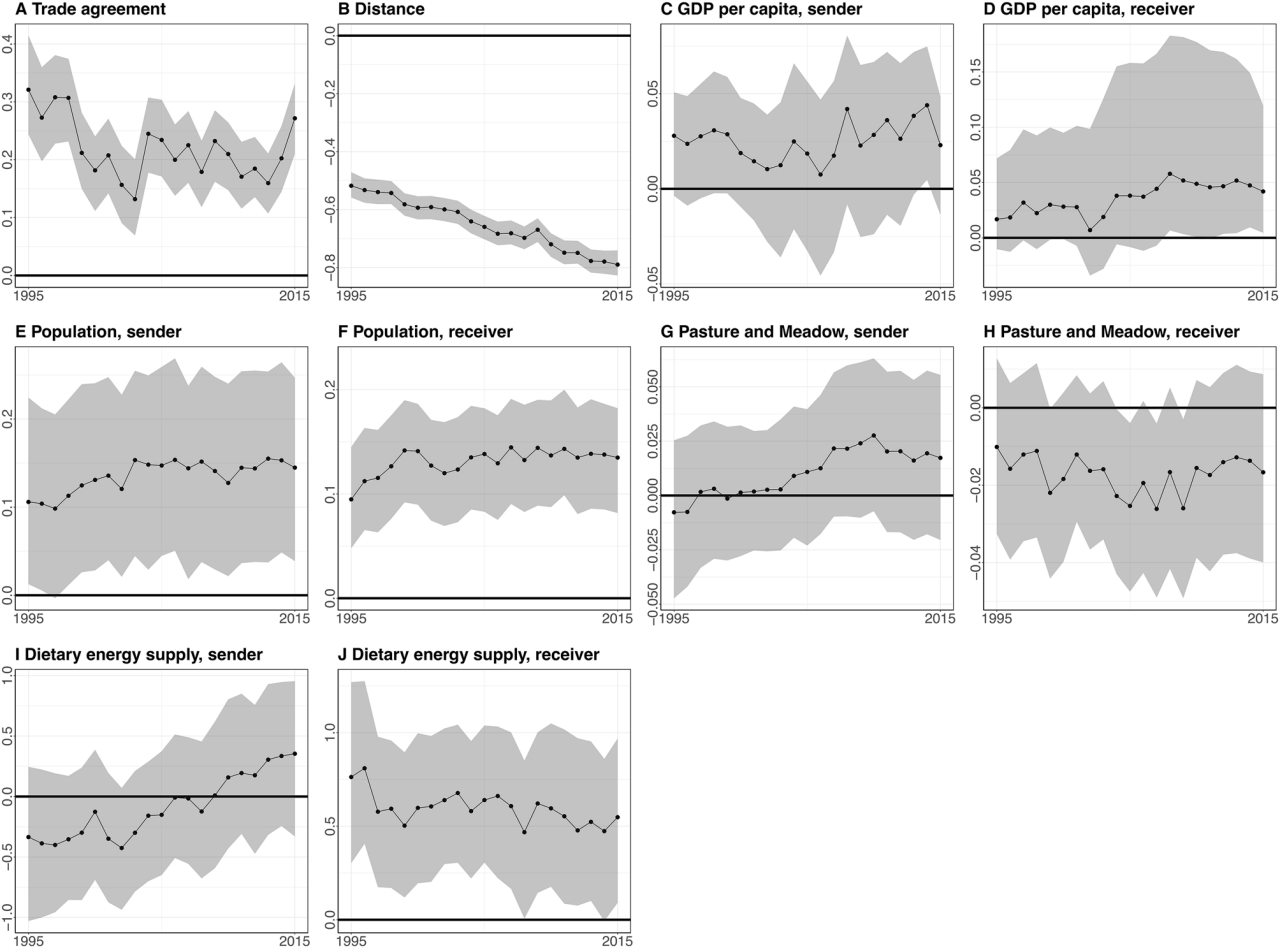
Mean and 95% credible intervals of the coefficients from 1995 to 2015: **(A) **the presence of trade agreements between sending and receiving countries (presence = 1), **(B)** geographic distance between sending and receiving countries (km), **(C) **per capita GDP in sending countries (const. 2010 US $), **(D)** per capita GDP in receiving countries (const. 2010 US $), **(E)** population size in sending countries (1000 persons), **(F)** population size in receiving countries (1000 persons), **(G) **the size of pastures and meadows in sending countries (km^2^), **(H) **the size of pastures and meadows in receiving countries (km^2^), **(I)** average dietary energy supply adequacy in sending countries (%), and **(J) **average dietary energy supply adequacy in receiving countries (%).

Results of the mixed-effects model showed the mean and 95% credible intervals for each coefficient from 1995 to 2015 (Fig. 3). First, the presence of trade agreements between countries was positively associated with the quantities of meat trade (Fig. 3A). The coefficients of trade agreement declined from 1995 to 2004 and then shifted upward from 2004 and 2015. Since there may be possible omitted variables that would invalidate our inferences, we calculated the robustness of our inference for an effect of trade agreement (in 1995 and 2015) on amount of meat trade^23^. To invalidate the inference of a positive effect of trade agreements on meat trade flows in 1995, we would have to replace 98% of the data (17,523 out of 17,822 pairs of countries in 1995) with null hypothesis cases in which there was no effect of trade agreements on the extent of trading. In 2015, the inference for a positive relationship of trade agreements with the meat trade flows is slightly more robust as 99% of our data (17,606 out of 17,822 pairs of countries in 2015) would have to be replaced with the null hypothesis cases (no effect of trade agreement on meat trade) to invalidate the inference. Although omitted variables may exist for our model, we believe our inferences are robust, even to the most plausible omitted variables.

Second, receiving countries tended to import more meat products from nearby countries. In other words, geographic distance between countries was negatively associated with the quantities of meat trade (Fig. 3B). The coefficients for geographic distance were increasingly negative over time, indicating a strengthening of this relationship.

Third, per capita GDP in receiving countries had a positive association with the quantities of meat imports after 2008 (Fig. 3D). While the credible intervals for per capita GDP contained zero from 1995 to 2007, they were positive from 2008 to 2015. Per capita GDP in sending countries had positive coefficients, but the credible intervals for this variable consistently contained zero (Fig. 3C).

Fourth, population size in both sending and receiving countries had a significant positive association with meat trade (Fig. 3E, F). However, there was more uncertainty associated with the effect of population size in sending countries than in receiving countries.

Fifth, while pastureland coefficients were positive for sending countries and negative for receiving countries, this relationship was not significant (Fig. 3G, H). Finally, countries with higher dietary energy supply tended to import more meat products (Fig. 3J), while dietary energy supply in sending countries was not significant throughout the study period (Fig. 3I).

The mixed-effects model also measured the auto-regressive impacts of the previous year on meat exports (Φ_s_), imports (Φ_r_), and their reciprocity in the present year (Table 1). The medians of Φ_s_ and Φ_r_ were 0.992 and 0.980, respectively. These results indicate that the quantities of meat exports in the present year depended substantially on the level of meat exports from the past year, and the same was true for meat imports. However, there was little evidence of previous imports affecting current exports and vice versa with median values of Φ_sr_ and Φ_rs_ at 0.015 and 0.003, respectively. The median of Φ_gg_ was 0.039, indicating that the degree of reciprocity between countries in the past year could not account for the level of reciprocity in the present year.

**Table 1:** Phi parameter estimates for the mixed-effects model with median and 95% quantile intervals.

Parameter	Median	2.5%	97.5%
${\Phi }_{s}$	0.992	0.966	0.995
${\Phi }_{r}$	0.980	0.449	0.985
${\Phi }_{sr}$	0.015	−0.007	0.030
${\Phi }_{rs}$	0.003	0.001	0.046
${\Phi }_{gg}$	0.039	0.037	0.041

## Discussion

This study represents the first time that a network model and cluster analysis have been combined in the analysis of global meat trade. The combined use of cluster analysis and mixed-effects modeling provides a comprehensive perspective of the dynamics of meat trade networks. Consistent with previous findings, population size in both sending and receiving countries significantly contributes to increases in meat trade from 1995 to 2015^1,2,10^. In receiving countries, per capita GDP has a positive impact on the quantities of meat imports after 2008, but these coefficients have high uncertainty. This result is most likely due to the increased role of meat imports in low- and middle-income countries such as China, Thailand, Cote d’Ivoire, and Republic of the Congo in addition to imports from high-income countries^24,25^.

Our results also provide evidence that international trade agreements contribute to changes in the structure of the global meat trade network. For example, we found that the coefficients of trade agreements slightly increased after 2004, the year in which global meat trade began to form consolidated networks. Trade agreements between sending and receiving countries can result in firms outsourcing labor-intensive stages of production to other countries and then re-importing those products back into the receiving countries^26^. The North American Free Trade Agreement, which went into effect in 1994, could explain the consolidation of the North American meat trade cluster^6^. From 1995 to 2015, European countries also maintained a robust single cluster for meat trade with the relatively large number of regional trade agreements and short geographic distances between countries. European tariffs on meat imports from the United States may help to explain the distinct nature of the United States and European meat trade clusters^27,28^. With such information on the impacts of trade policy on trade network structure and development, policy makers can better understand and implement efficient cross-border efforts to foster sustainable food trade.

Unlike Europe and the United States, China has experienced dynamic cluster changes over time that are most likely associated with development and policy implementation processes. Throughout the latter half of the twentieth century, China was a net exporter of non-ruminant meat products (e.g., pork)^24,29^. Around the new millennium, increasing domestic meat demand as a result of rapid population and income growth caused China to switch to a net importer^24,29^. Furthermore, China’s Belt and Road development initiative may have influenced the cluster shift from the North America-based group to the Asia- and South America-based group after 2014 (Supplementary Fig. 1). This change makes sense given that the objectives of the Belt and Road Initiative are to stimulate trade and economic growth by building a consolidated market with Asia, Latin America, Africa, and Europe via land and marine networks^30^. As China has signed new trade agreements with Asian and European countries participating in the Belt and Road Initiative, these trade agreements may be a cause of China’s cluster shift to new meat trade partners^31,32^.

In addition, our results indicate that countries with high food availability tend to import substantial amounts of meat products from abroad. In other words, although many countries fulfill their dietary energy requirements, they import meat products to satisfy consumers’ various meat preferences that are not produced domestically due to limited agricultural land and/or a large population^6,10,16^. Elevated levels of meat imports in countries with increased food availability may enable diets high in meat and thus increase diet-related, non-communicable diseases^10–12^.

From our network model results, the non-significant but consistent positive coefficients for sending countries and negative coefficients for receiving countries’ pastureland lends some support to our hypothesis that meat is traded from areas of greater pastureland to areas with less pastureland^10^. Furthermore, meat production for exports may indirectly affect the environment and biodiversity through land use changes for animal feed production^7–9^. For example, soybean trade that is used for meat production in some soy-importing countries (e.g., China) may negatively impact the environment in countries sending soy (e.g., Brazil) because many sending countries convert natural habitats to agricultural lands for soybean exports^5,20,33^. On the other hand, the environment in importing countries may also be negatively affected due to land use change as a result of imports^34^.

While the results of our study predominantly support our hypotheses, there are a few limitations that should be considered when interpreting the results of this analysis. First, it is challenging to determine the most critical factors in our longitudinal network analyses as each variable fluctuates within a country over time and/or among countries during the same time period. Second, our study concentrated on global meat trade networks, but many countries are also highly dependent on animal feed imports for increasing domestic meat production. Future research will be necessary to adopt our integrated network approach to animal feed trade across countries and incorporate it within our meat trade results. Third, our network model could not include several factors that potentially influence meat trade flows due to the lack of global time-series data. For example, although some global crisis events (e.g., financial crisis and animal diseases) may change the structure of meat trade networks regionally and globally, we could not quantify such relationships using our network model, due to the lack of comprehensive data regarding global crisis events across countries and over time. Despite these limitations, this study provides a new integrated network approach to simultaneously examine the structural changes in meat trade networks and their crucial influencing factors.

Our network results provide strategic insights to better understand the unintended social and environmental consequences of increasing global meat trade. Identifying the countries that are responsible for increases in meat trade and the underlying forces driving these changes can help stakeholders and policy makers to implement efficient cross-border efforts for conserving natural habitats in exporting countries and mitigating diet-related human health risks in importing countries simultaneously. In conclusion, our approach and findings contribute a better groundwork for the implementation of sustainable meat trade in pursuit of improved human health and nature conservation.

## Methods

### Data collection

Data on global meat trade were obtained from the Food and Agriculture Organization (FAO) food trade matrix dataset^35^. We included 14 red meat and six processed meat items, primarily from beef and pork (Supplementary Table 1). Within the FAO trade matrix dataset, we used the meat export matrix data. Using R, we first filled out the bilateral meat trade matrix by using the FAO export matrix data, and then we used the FAO import matrix data to fill data gaps in the bilateral meat trade matrix. The annual total meat trade (in metric tons) between countries from 1995 to 2015 was used for all subsequent analyses. The physical volume of meat trade was chosen over the monetary value of meat as it is more appropriate to link with meat production activities and human health impacts of meat consumption. The monetary value varies with price elasticity and thus has a risk to underestimate low-priced products (e.g., India’s buffalo meat).

We also collected data regarding possible drivers of global meat trade between countries. Over the period from 1995 to 2015, these factors consisted of political, demographic, economic, environmental, and geographical features in sending countries, receiving countries, and country pairs. At the level of country pairs, trade agreement data were obtained from the World Trade Organization (WTO)^15^. The WTO data are the best available global trade agreement dataset, as the WTO is the only international organization, operating a system of trade rules between countries. We did not include trade agreements only for services as this study focused on global meat trade networks. Additionally, we calculated geographic distances between the centroids of countries by using GeoDa^36^. Geographic distance remained a constant variable throughout our study period.

In both sending and receiving countries, per capita gross domestic product (GDP) and population size were obtained from the World Bank^37^. Per capita GDP and population size can be interpreted as relative income level and market size, respectively. We also collected data regarding the size of permanent pastures and meadows in agricultural lands^35,37^. The index of average dietary energy supply adequacy was obtained to represent food availability in each country^35^. This indicator measures the proportion of average supply for food consumption to the dietary energy requirement in each country. Countries with values less than 100% experience a lack of food supply.

We excluded countries when they had missing data values for the explanatory variables at any time over the 1995 to 2015 duration. For bilateral meat trade data, we also excluded countries from the entire network analysis when their aggregated volume of meat trade was zero at any point from 1995 to 2015. Of the 176 countries from which explanatory variable data were available, we removed 42 countries from the mixed effects model due to missing data. These included several smaller countries with inconsistent meat trade patterns (e.g., Bahrain and Maldives). The final dataset for the network analyses contained 134 countries from 1995 to 2015.

### Cluster analysis

We used the optimal community detection algorithm in R to identify clusters of countries in the global meat trade network. Meat trade networks consist of ties (edges) among actors (nodes or vertices). For this cluster analysis, the meat trade data included annual meat trade quantities between each pair of 134 countries from 1995 to 2015. The quantity of meat trade is a directed flow from sending country to receiving country. The optimal community algorithm generates clusters that maximize the modularity score across all possible clusters^38^. Countries in the same cluster tend to trade more meat products with each other than those in different clusters. Thus, the optimal community algorithm helps identify clusters within the global meat trade network and allows us to examine whether these clusters are based on geographic location or other factors, such as income level and population size. This optimal community algorithm had the highest modularity among community detection algorithms developed for directed flows (Supplementary Table 2). We also compared with different algorithms to identify the robustness of our cluster results (Supplementary Table 3). Specifically, we selected the walktrap algorithm for the comparison, as this algorithm had the second-highest modularity among different algorithms. The walktrap algorithm generates densely connected clusters of a given network by measuring structural similarity between actors via random walks^39^. Networks with high modularity have dense ties between actors in the same clusters but sparse ties with actors of different clusters^38^. We used the *igraph* package in R to visualize the cluster results^22^. In the graphs, we used the k-core decomposition approach to examine the core and peripheral countries in global meat trade networks. In a given network, the k-core decomposition approach consecutively eliminates the least connected nodes based on bilateral meat trade flows and thus mainly allows for identifying the central countries for a network figure^40^. Then, we demonstrated the cluster results by country on the world map using ArcGIS^41^.

### Hypothesized factors for global meat trade

Following previous studies that investigated factors shaping global meat trade, we incorporated the most commonly used factors into our network analysis. We note that the factors used in meat trade models may change largely depending on research questions, data availability, methodologies, and selection of countries and time periods. These factors represent political, demographic, economic, and environmental factors that have been shown to influence meat trade networks. First, the presence of trade agreements between sending and receiving countries has been found to be positively associated with meat trade quantities due to economic benefits (e.g., low tariffs)^12,42–44^. Trade agreements can contribute to promoting meat trade with tariff reductions and the liberalization of non-tariff barriers such as food safety standards and domestic regulations^45,46^. Second, countries may be metacoupled (e.g., through trade between adjacent countries and between distant countries)^47^, and a shorter distance between sending and receiving countries plays a crucial role in increasing meat trade^6^. Distance has also been shown to be very important in the trade of other products^48,49^, as well as in other human activities such as global fishing^50^, tourism^51–53^, and flows of ecosystem services^54–56^. Third, income level and population size have been found to be important determinants of global meat trade^1,2^. However, these factors act differently for meat trade in sending and receiving countries. In both sending and receiving countries, population size has been positively associated with meat trade, while in receiving countries income has also been significantly positively associated with meat trade^10^. Fourth, pastures and meadows in agricultural lands affect meat production for exports as pasturelands are essential for cattle ranching and animal feed^7–9,57^. Countries with less pastureland may depend on meat imports from countries with more pastureland. Fifth, countries with high food availability for human consumption tend to have both high meat exports and imports^10,16^. Substantial domestic meat supply is essential for the availability of meat products for export^58^. Many countries with high food availability also depend on meat imports to meet demands for a meat-rich diet^16^.

### Mixed effects model

Over the last two decades, many network models have been developed to explain network dependencies^17^. For instance, many networks depend on the earlier structure of network ties to form their current ties, a process referred to as temporal dependence^17,59,60^. Conventional statistical models (e.g., ordinary least squares) that do not evaluate these dependencies risk overestimation of parameters’ significance. The mixed effects model extends upon previous models by explaining both network and temporal dependencies^61^ through the use of a latent space approach to account for sender, receiver, and reciprocity effects^17,59,62^. This model assumes the existence of latent variables to visualize network structures within the latent space positions and uses the framework for a generalized linear model that allows for adoption of the gravity approach.

We used a mixed effects model for quantifying longitudinal meat trade networks. The dependent variable was the amount of meat trade flows from an exporting country to an importing country. This mixed effects model, developed by Westveld and Hoff^61^, accounts for both network and temporal dependencies as well as the influence of external variables in the global meat trade network. For the mixed effects model, we did not exclude zero-valued observations and also filled in zeros where pairs of countries have missing values in bilateral meat trade data. Given that all the countries included in the analysis reported at least some meat trade data, assuming a zero value for unreported trade connections is reasonable. Additionally, eliminating or neglecting zero-trade values could result in the loss of important information regarding low levels of meat trade^63^, and could also lead to biased results when these zero values have a non-random distribution within the network^64,65^.

Variables included in the mixed effects model were selected through the gravity approach. In the gravity model’s assumption, the quantities of meat trade between two countries increase with a country’s economic and demographic size and decrease with transportation costs between countries^65–67^. The gravity model can also include some characteristics and policies regarding meat trade flows in sending and receiving countries^66^. Previous analyses have used the gravity approach to examine meat trade; however, applications of the gravity approach to meat trade have not yet been conducted on a global scale^66,68^.

We implemented the mixed effects model for annual meat trade networks between 134 countries for each year from 1995 to 2015 using the MCMC package in R^22^. The parameters of the mixed effects model are estimated with a Bayesian inference approach based on the Markov-Chain Monte Carlo (MCMC) algorithm^61^. Our mixed effects model was based on R code script from Westveld and Hoff^61^. The model for longitudinal meat trade flows is shown as:

$$\mathrm{ln}{\mathrm{Meat\, trade}}_{\mathrm{ijt}}={\upbeta }_{0\mathrm{t}}+{\upbeta }_{1\mathrm{t}}{\mathrm{Trade\, agreement}}_{\mathrm{ijt}}+{\upbeta }_{2\mathrm{t}}{\mathrm{lnDistance}}_{\mathrm{ij}}+{\upbeta }_{3\mathrm{t}}\mathrm{ln}{\mathrm{GDP\, per\, capita}}_{\mathrm{it}}+{\upbeta }_{4\mathrm{t}}\mathrm{ln}{\mathrm{GDP\, per\, capita}}_{\mathrm{jt}}+{\upbeta }_{5\mathrm{t}}{\mathrm{lnPopulation}}_{\mathrm{it}}+{\upbeta }_{6\mathrm{t}}{\mathrm{lnPopulation}}_{\mathrm{jt}}+{\upbeta }_{7\mathrm{t}}\mathrm{ln}{\mathrm{Pasture \,land}}_{\mathrm{it}}+{\upbeta }_{8\mathrm{t}}\mathrm{ln}{\mathrm{Pasture\, land}}_{\mathrm{jt}}+{\upbeta }_{9\mathrm{t}}\mathrm{ln}{\mathrm{Dietary \,energy\, supply}}_{\mathrm{it}}+{\upbeta }_{10\mathrm{t}}\mathrm{ln}{\mathrm{Dietary\, energy \,supply}}_{\mathrm{jt}}+{\mathrm{s}}_{\mathrm{it}}+{\mathrm{r}}_{\mathrm{jt}}+{\mathrm{g}}_{\mathrm{ijt}}$$

where: Meat trade_ijt_ is the quantity of meat trade from a sending country i to a receiving country j at time t; Trade agreement_ijt_ is the presence of trade agreement between the sending and receiving countries at time t; Distance_ij_ is the geographic distance between the centroid of country i and the centroid of country j; GDP per capita_it_ and GDP per capita_jt_ denote per capital GDP in the sending and receiving countries at time t, respectively; Population_it_ and Population_jt_ are population size in the sending and receiving countries at time t, respectively; Dietary energy supply_it_ and Dietary energy supply_jt_ represent the food availability in sending and receiving countries at time t; s_it_ is a sender effect; r_jt_ is a receiver effect; g_ijt_ is a residual error term; s_it_ and r_jt_ are the country-specific random effects of sender and receiver, which estimate the average deviations regarding the levels of meat exports and imports in each country. These effects help identify the most or least active countries in global meat trade networks.

Using the MCMC algorithm, we estimated model parameters over 11,000 iterations with the first 1000 iterations dropped in order to assure convergence toward the stationary distribution. We saved results from every 10th iteration. Then we calculated means and 95% credible intervals of our independent variables by using the joint posterior distribution. We computed the 95% credible intervals with the Highest Posterior Density interval in R^22^. When the credible intervals contain zero, the coefficients are not statistically significant at the p=0.05 level.

Our global-level network analyses consisted of cluster analyses and a statistical network model. The cluster analyses allowed us to determine changes in the network structure of meat trade from 1995 to 2015. Our network model examined which factors contribute to increases in meat trade flows over time while quantifying network and temporal dependencies in global meat trade networks.

We also recognize that our inference regarding an effect of independent variables on the amounts of meat trade could be biased due to variables that were omitted from the analysis. Although we made efforts to identify key variables and include them in our model, there may still be possible omitted variables affecting any inferences about factors affecting the amount of meat trade. In response to concerns about omitted variable bias, we examined the robustness of our inferences using the sensitivity analysis developed by Frank et al*.*^23^. This sensitivity analysis quantifies the degree of bias that there must be in an estimated effect to invalidate an inference. It can be interpreted as the proportion of cases that would have to be replaced with null hypothesis cases to invalidate an inference^23^. Our threshold for statistical significance was the conventional α=0.05. We also used the time-series standard error for the sensitivity analysis, as the time-series standard error accounts for possible autocorrelation in the MCMC samples.

## Supplementary information

Supplementary Information.

## Data Availability

All datasets and computer codes used in conducting the analyses summarized in this paper are available from the corresponding author.
