# Supplementary material for: The spatial and temporal dynamics of global meat trade networks
Source: Sci Rep. 2020 Oct 7;10:16657. doi: 10.1038/s41598-020-73591-2 (PMC7541524; doi:10.1038/s41598-020-73591-2)
Supplement: Supplementary file 1 — Supplementary Information. [file 41598_2020_73591_MOESM1_ESM.docx]

**Supplementary Information**

**Supplementary Table 1.** Meat items for network analyses

| **Red meat**, 14 items |
| --- |
| Meat, Cattle; Offals of cattle; Meat of cattle, boneless; Meat of sheep; Offals of sheep; Goat meat; Offals of goats; Pig meat; Offals of pigs; Pork; Horse meat; Meat of asses; Rabbit meat; Game meat |
| **Processed meat**, 6 items |
| Sausages of Beef and Veal; Beef and Veal Preparations nes; Bacon and Ham; Sausages of Pig Meat; Pig Meat Preparations; Meat, Dried |

**Supplementary Table 2.** The number of clusters and modularity scores across different community algorithms.

| **community algorithm** | **1995** | | **2015** | |
| --- | --- | --- | --- | --- |
|  | **cluster number** | **modularity** | **cluster number** | **modularity** |
| optimal.community | 8 | 0.408 | 4 | 0.424 |
| walktrap.community | 17 | 0.389 | 11 | 0.411 |
| infomap.community | 19 | 0.353 | 10 | 0.331 |
| leading.eigenvector.community | 5 | 0.054 | 24 | 0.392 |
| edge.betweenness.community | 14 | <0.001 | 68 | 0.017 |
| springlass.community | 10 | <0.001 | 7 | <0.001 |

**Supplementary Table 3**. The comparisons of clusters between optimal community algorithm and walktrap community algorithm in 1995 and 2015.

| **County** | **1995** | | **County** | **2015** | |
| --- | --- | --- | --- | --- | --- |
|  | **Optimal** | **Walktrap** |  | **Optimal** | **Walktrap** |
|  | 0.408 | 0.389 |  | 0.424 | 0.411 |
| Albania | 1 | 1 | Austria | 1 | 1 |
| Austria | 1 | 1 | Bulgaria | 1 | 1 |
| Azerbaijan | 1 | 1 | Bosnia and Herzegovina | 1 | 1 |
| Benin | 1 | 1 | Central African Republic | 1 | 1 |
| Burkina Faso | 1 | 1 | Switzerland | 1 | 1 |
| Bosnia and Herzegovina | 1 | 1 | Côte d’Ivoire | 1 | 1 |
| Côte d’Ivoire | 1 | 1 | Cameroon | 1 | 1 |
| Cameroon | 1 | 1 | Congo | 1 | 1 |
| Congo | 1 | 1 | Cyprus | 1 | 1 |
| Cabo Verde | 1 | 1 | Czechia | 1 | 1 |
| Cyprus | 1 | 1 | Germany | 1 | 1 |
| Czechia | 1 | 1 | Denmark | 1 | 1 |
| Germany | 1 | 1 | Spain | 1 | 1 |
| Denmark | 1 | 1 | Estonia | 1 | 1 |
| Algeria | 1 | 1 | Finland | 1 | 1 |
| Egypt | 1 | 1 | France | 1 | 1 |
| Spain | 1 | 1 | United Kingdom | 1 | 1 |
| France | 1 | 1 | Ghana | 1 | 1 |
| Gabon | 1 | 1 | Greece | 1 | 1 |
| United Kingdom | 1 | 1 | Croatia | 1 | 1 |
| Georgia | 1 | 1 | Hungary | 1 | 1 |
| Ghana | 1 | 1 | Ireland | 1 | 1 |
| Guinea | 1 | 1 | Iceland | 1 | 1 |
| Greece | 1 | 1 | Italy | 1 | 1 |
| Ireland | 1 | 1 | Lithuania | 1 | 1 |
| Iran | 1 | 1 | Latvia | 1 | 1 |
| Italy | 1 | 1 | Morocco | 1 | 1 |
| Lebanon | 1 | 1 | North Macedonia | 1 | 1 |
| Morocco | 1 | 1 | Mali | 1 | 1 |
| Madagascar | 1 | 1 | Malta | 1 | 1 |
| Malta | 1 | 1 | Netherlands | 1 | 1 |
| Namibia | 1 | 1 | Norway | 1 | 1 |
| Nigeria | 1 | 1 | Poland | 1 | 1 |
| Netherlands | 1 | 1 | Portugal | 1 | 1 |
| Portugal | 1 | 1 | Romania | 1 | 1 |
| Senegal | 1 | 1 | Senegal | 1 | 1 |
| Slovakia | 1 | 1 | Suriname | 1 | 1 |
| Eswatini | 1 | 1 | Slovakia | 1 | 1 |
| Togo | 1 | 1 | Slovenia | 1 | 1 |
| Tunisia | 1 | 1 | Sweden | 1 | 1 |
| Zambia | 1 | 1 | Togo | 1 | 1 |
| Zimbabwe | 1 | 1 | Guinea | 1 | 3 |
| Guyana | 1 | 2 | Sao Tome and Principe | 1 | 10 |
| Malawi | 1 | 5 | Australia | 2 | 2 |
| Rwanda | 1 | 13 | Belize | 2 | 2 |
| Sierra Leone | 1 | 14 | Barbados | 2 | 2 |
| Australia | 2 | 2 | Canada | 2 | 2 |
| Belize | 2 | 2 | Costa Rica | 2 | 2 |
| Barbados | 2 | 2 | Cuba | 2 | 2 |
| Brunei Darussalam | 2 | 2 | Fiji | 2 | 2 |
| Canada | 2 | 2 | Gambia | 2 | 2 |
| Colombia | 2 | 2 | Guatemala | 2 | 2 |
| Costa Rica | 2 | 2 | Honduras | 2 | 2 |
| Cuba | 2 | 2 | Indonesia | 2 | 2 |
| Ecuador | 2 | 2 | Jamaica | 2 | 2 |
| Estonia | 2 | 2 | Japan | 2 | 2 |
| Fiji | 2 | 2 | Cambodia | 2 | 2 |
| Honduras | 2 | 2 | Republic of Korea | 2 | 2 |
| Indonesia | 2 | 2 | Sri Lanka | 2 | 2 |
| Jamaica | 2 | 2 | Mexico | 2 | 2 |
| Jordan | 2 | 2 | Mauritius | 2 | 2 |
| Japan | 2 | 2 | Malaysia | 2 | 2 |
| Kenya | 2 | 2 | Nicaragua | 2 | 2 |
| Republic of Korea | 2 | 2 | New Zealand | 2 | 2 |
| Sri Lanka | 2 | 2 | Panama | 2 | 2 |
| Mexico | 2 | 2 | Solomon Islands | 2 | 2 |
| Mauritius | 2 | 2 | El Salvador | 2 | 2 |
| Malaysia | 2 | 2 | Trinidad and Tobago | 2 | 2 |
| Niger | 2 | 2 | United States of America | 2 | 2 |
| New Zealand | 2 | 2 | Saint Vincent and the Grenadines | 2 | 2 |
| Pakistan | 2 | 2 | Vanuatu | 2 | 2 |
| Panama | 2 | 2 | Colombia | 2 | 3 |
| Philippines | 2 | 2 | Jordan | 2 | 3 |
| Saudi Arabia | 2 | 2 | Bangladesh | 2 | 4 |
| Solomon Islands | 2 | 2 | Oman | 2 | 4 |
| Thailand | 2 | 2 | Saudi Arabia | 2 | 4 |
| Trinidad and Tobago | 2 | 2 | Yemen | 2 | 4 |
| United States of America | 2 | 2 | Guyana | 2 | 6 |
| Saint Vincent and the Grenadines | 2 | 2 | Albania | 3 | 1 |
| Venezuela | 2 | 2 | Cabo Verde | 3 | 1 |
| Vanuatu | 2 | 2 | Gabon | 3 | 1 |
| Yemen | 2 | 2 | Ecuador | 3 | 2 |
| Guatemala | 2 | 6 | Argentina | 3 | 3 |
| Nicaragua | 2 | 6 | Armenia | 3 | 3 |
| El Salvador | 2 | 6 | Azerbaijan | 3 | 3 |
| Afghanistan | 2 | 7 | Belarus | 3 | 3 |
| Bangladesh | 2 | 9 | Bolivia | 3 | 3 |
| Kyrgyzstan | 2 | 11 | Brazil | 3 | 3 |
| Cambodia | 2 | 12 | Chile | 3 | 3 |
| Belarus | 3 | 1 | China | 3 | 3 |
| Finland | 3 | 1 | Egypt | 3 | 3 |
| Croatia | 3 | 1 | Georgia | 3 | 3 |
| Hungary | 3 | 1 | Iran | 3 | 3 |
| Iceland | 3 | 1 | Israel | 3 | 3 |
| Lithuania | 3 | 1 | Kazakhstan | 3 | 3 |
| Latvia | 3 | 1 | Kyrgyzstan | 3 | 3 |
| Norway | 3 | 1 | Lebanon | 3 | 3 |
| Poland | 3 | 1 | Republic of Moldova | 3 | 3 |
| Sao Tome and Principe | 3 | 1 | Mongolia | 3 | 3 |
| Slovenia | 3 | 1 | Peru | 3 | 3 |
| Sweden | 3 | 1 | Paraguay | 3 | 3 |
| United Republic of Tanzania | 3 | 1 | Russian Federation | 3 | 3 |
| China | 3 | 2 | Tunisia | 3 | 3 |
| Kazakhstan | 3 | 2 | Ukraine | 3 | 3 |
| Mongolia | 3 | 2 | Uruguay | 3 | 3 |
| Russian Federation | 3 | 2 | Venezuela | 3 | 3 |
| Uganda | 3 | 2 | Madagascar | 3 | 7 |
| Bulgaria | 3 | 4 | Benin | 4 | 1 |
| Central African Republic | 3 | 4 | Burkina Faso | 4 | 2 |
| Republic of Moldova | 3 | 4 | Nepal | 4 | 3 |
| North Macedonia | 3 | 4 | Philippines | 4 | 3 |
| Romania | 3 | 4 | Thailand | 4 | 3 |
| Ukraine | 3 | 4 | Afghanistan | 4 | 4 |
| Armenia | 3 | 8 | United Arab Emirates | 4 | 4 |
| Gambia | 3 | 10 | Brunei Darussalam | 4 | 4 |
| Switzerland | 4 | 1 | Algeria | 4 | 4 |
| Argentina | 4 | 3 | Ethiopia | 4 | 4 |
| Bolivia | 4 | 3 | India | 4 | 4 |
| Brazil | 4 | 3 | Kenya | 4 | 4 |
| Chile | 4 | 3 | Kuwait | 4 | 4 |
| Israel | 4 | 3 | Niger | 4 | 4 |
| Peru | 4 | 3 | Pakistan | 4 | 4 |
| Paraguay | 4 | 3 | Sierra Leone | 4 | 4 |
| Uruguay | 4 | 3 | United Republic of Tanzania | 4 | 4 |
| Suriname | 4 | 15 | Botswana | 4 | 5 |
| Kuwait | 5 | 2 | Namibia | 4 | 5 |
| United Arab Emirates | 5 | 5 | Nigeria | 4 | 5 |
| India | 5 | 5 | Eswatini | 4 | 5 |
| Mali | 5 | 5 | South Africa | 4 | 5 |
| Oman | 5 | 5 | Zambia | 4 | 5 |
| Botswana | 6 | 1 | Zimbabwe | 4 | 5 |
| South Africa | 6 | 2 | Malawi | 4 | 8 |
| Ethiopia | 7 | 18 | Rwanda | 4 | 9 |
| Nepal | 8 | 19 | Uganda | 4 | 11 |

**Supplementary Figure 1.** Clusters of global meat trade networks by country: (A) 1995, (B) 1996, (C) 1997, (D) 1998, (E) 1999, (F) 2000, (G) 2001, (H) 2002, (I) 2003, (J) 2004, (K) 2005, (L) 2006, (M) 2007, (N) 2008, (O) 2009, (P) 2010, (Q) 2011, (R) 2012, (S) 2013, (T) 2014, and (U) 2015. Countries in the same cluster (or color) tend to trade more meat products with each other than those in different clusters. The map was generated by ArcGIS 10.3^41^.
